# Three steps to maintain predictable interdental papilla and gingiva emergence profiles in immediate implant placement. A 3-year follow-up case report

**DOI:** 10.4317/jced.54863

**Published:** 2018-05-01

**Authors:** Ramón Gómez-Meda, Vanessa Montoya-Salazar, Santiago Dalmau, Daniel Torres-Lagares

**Affiliations:** 1 DDS, MS. School of Dentistry. University of Seville, Spain; 2 PhD, DDS. Master in Oral Surgery. School of Dentistry. University of Seville, Spain; 3 Technician in Dental Prostheses. Madrid, Spain; 4 PhD, DDS, MSc (Oral Surgery). Professor of Oral Surgery. Department of Stomatology. University of Seville, Spain

## Abstract

We present a case that describes a three-step clinical technique to provide guidelines to replace fractured teeth with immediate implant placement using the same dental structure as a temporary crown and a regenerative approach. This approach predictably maintains the interdental papilla and gingiva emergence profile to ensure a favorable cosmetic result. A 3-year follow-up has shown good clinical outcomes and stability in crestal bone levels. Consequently, this is an innovative way to do temporary crown and design restorations in everyday clinical practice.

** Key words:**Interdental papilla, dental implant, inmmediate implant, gingival aesthetics, dental aesthetics.

## Introduction

Dental fractures can occur in endodontically treated teeth even when restored with glass-fiber reinforced posts and cast gold posts (1). After extraction, maintenance of the interdental papilla remains one of the most challenging goals for clinicians (2). When a fractured single tooth is replaced with an immediate implant, the papilla between the neighboring natural tooth and implant can often be maintained or predictably reformed as long as the adjacent tooth’s periodontal attachment and bone are preserved (2).

Many studies have reported on the clinical outcomes of immediate implants inserted in postextraction sockets (3-5). A one-step surgical procedure reduces treatment time, improves aesthetic outcomes, increases comfort during healing, and has proven to be a predictable strategy with a high success rate in areas with or without periapical lesions. In contrast with the traditional protocol, the immediate placement of an implant after tooth extraction also maintains the horizontal and vertical dimensions of the osseous tissues and keeps the implants at the same angulation as the pre-existing natural teeth (3-5). Soft tissue augmentation procedures may be indicated for the increase of soft tissue thickness and keratinized tissue, the reduction of interproximal peri-implant bone loss, and the coverage of shallow peri-implant soft tissue recessions. Also, bone-regeneration approaches showed efficacy when used for ridge reconstruction after complete healing of the soft tissues, and the stability of the augmented bone may play a role in the maintenance of the soft tissue position and dimensions (6).

CAD/CAM technology is especially helpful in postextraction implant for aesthetic rehabilitation, as it is possible to immediately fix a provisional crown with an anatomic shape that allows an optimal healing process of the tissues (7). Immediate provisionalization also has the advantage of preserving the shape of the soft tissues and patients’ well-being and self-steem (7). Most clinicians have used zirconia abutments for aesthetic reasons, which have shown the longest survival in external connection, whereas the internal zirconia connection showed the highest fracture incidence over the observation period (8). Zirconia abutments showed satisfactory clinical performance in the anterior and posterior regions after 4 to 10 years. The restoration of vertical height and connection type influenced the clinical longevity of restorations; in particular, internal connections with secondary metallic components reduced the incidence of complications (6,9,10).

Ceramics preclude direct interaction between zirconia and soft tissue cells, thus reducing biocompatibility and benefit to the patient when zirconia is exposed to the tissues and no veneering porcelain is located below the gingival margin. Other authors have studied the impact of this treatment on soft peri-implant tissues after 3 years of follow-up. Soft tissue recession, vestibular contour, bleeding on probing, and probing depth were evaluated (6). No significant differences were observed between titanium and zirconia abutments when evaluating probing pocket depth, bleeding on probing, marginal bone levels, and mucosal recessions, but zirconia abutments demonstrated superiority in terms of achieving natural soft tissue color (6).

This article presents a 3-year follow-up for one single-tooth implant case in the premolar area that uses a regenerative approach with soft tissue augmentation to maintain gingiva emergence profile contours in just three steps.

## Case Report

**Figure 1:**
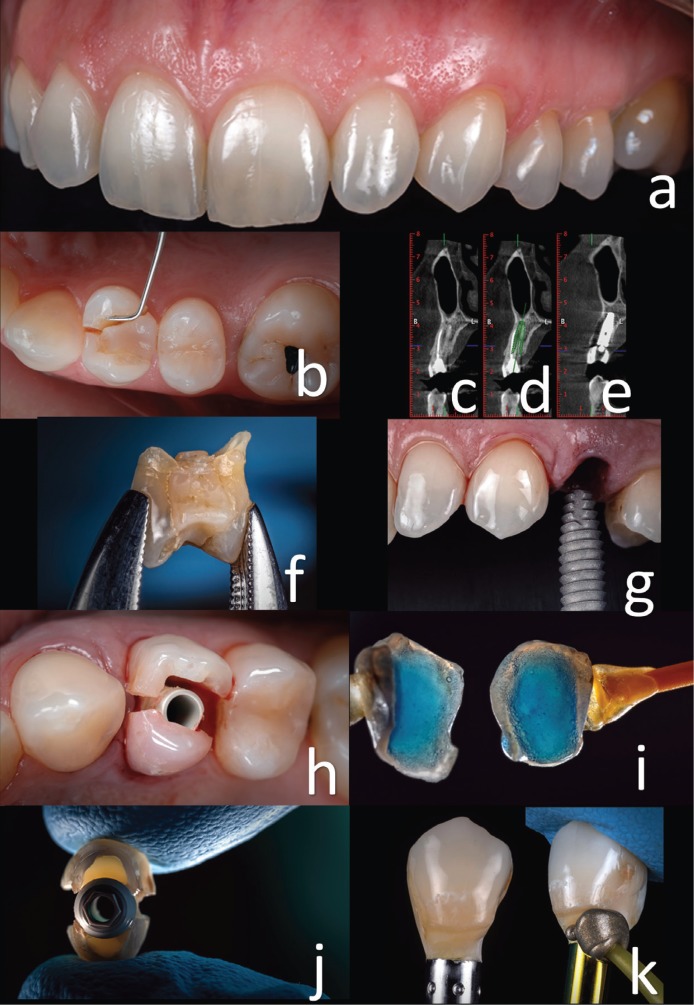
a) Buccal view, first premolar broken. b) Initial intraoral situation showing the vertical fracture in the endodontically treated tooth. c,d,e) Tomographic sequence treatment: initial, planning, and after implant and temporary crown placement. f) Crown tooth to fabricate the temporary crown. g) Implant placement. h) Temporary abutment with the hollowed crown tooth. i) Etched and rinsed. j) The temporary abutment is bonded. k) Finished crown with composite, polishing, and shining of the surface is advisable.

The case reported in the present article illustrates a therapeutic plan consisting of atraumatic extraction of the first premolar followed by immediate implant placement and immediate loading using the same fractured crown tooth in a 47-year-old female patient. The pre-operative situation showing the vertical fracture in an endodontically treated tooth was made years ago (Fig. 1a,b) and reported pain during biting and chewing. Tridimensional diagnostic data and dedicated software were used for treatment planning, allowing the achievement of optimal results (Fig. 1c,d,f) (3).

First step: Implant placement, maintenance of gingiva emergence profile contours, and connective tissue grafting.

**Figure 2:**
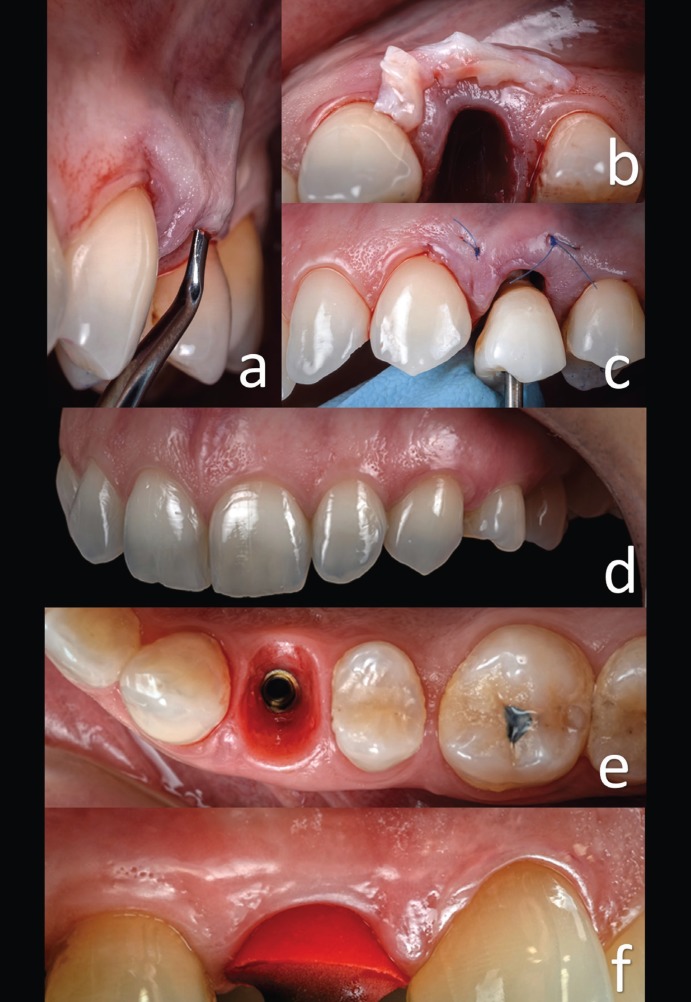
a) Preparing to allocate the connective tissue graft. b) Connective tissue graft from the tuberosity. c) Temporary crown screwed and the occlusion checked. d) Appearance after healing period. e) Emergence profile before impression. f) Gingiva profile is preserved in the vertical and horizontal dimensions. The individualized impression coping fits exactly in the shape of the gingiva.

Patient were asked to rinse two times before dental extraction with chlorhexidine 0.12% mouth rinse for a total of 1 minute. The surgery was performed under local anesthesia obtained by infiltration with articaine with adrenaline at a ratio of 1:100,000 (3). The surgical procedure was flapless. The tooth was gently extracted, with care taken not to damage the remaining socket walls, particularly the buccal wall. The crown of the extracted tooth is recovered to be used as provisional crown (Fig. 1g). The integrity of the residual walls of the alveolus was verified with a periodontal probe, and the procedure continued with the preparation of the implant site. The area was prepared following the standard protocol for implant placement, and the site preparation was extended apically 3-4 mm to achieve primary stability for the implants (following the instructions of the manufacturer) (3,5,11). The surgeon proceeded with the osteotomy, starting with a 2.0-mm-diameter pilot drill, to the desired depth. The bone quality of the residual crest was assessed according to the clinician’s judgment, and the implant site was prepared accordingly.4 Moderate modifications of the socket were accomplished at this stage to establish a better position and angulation of the implants. Thereafter, the endosseous titanium dental implant of 4.6 mm diameter with a platform–switching design and laser-lock technology was placed (Biohorizons Tapered Implant Plus, Biohorizons, U.S.A. ) 2 mm under the crestal level (3-5). The stability of the implants was determined clinically as the absence of axial or rotational movement. Immediately after implant placement, a prefabricated temporary abutment was prepared and screwed onto the implant. The hollowed crown of the tooth was used as temporary crown (Fig. 1h), and the internal part of the crown was etched and rinsed (Fig. 1i). The internal walls were bonded on the temporary abutment (Fig. 1j). The relining was done with light-curing resin composite (Gradia Anterior, GC, JAPAN). The provisional crowns were finished and polished meticulously to obtain the desired emergence profile (Fig. 1k). The emergence profile was replicated with silicone. This technique is used to maintain and transfer the emergence profile contours of a temporary crown to a definitive impression. This step will allow us to individualize an impression coping for the second appointment (12). A connective tissue graft from tuberosity was placed between the buccal bone plate and the gingiva to compensate for the collapse after remodeling residual bone (Fig. 2a,b) (13,14). Temporary restoration was screwed and the occlusion checked (Fig. 2c). The donor site was the maxillary tuberosity because this area provides grafts of distinct geometric shapes and histologic composition (13). Healing follow-up was performed 7 and 14 days after implant placement (Fig. 2d).

Second step: Reproducing emergency profile

**Figure 3:**
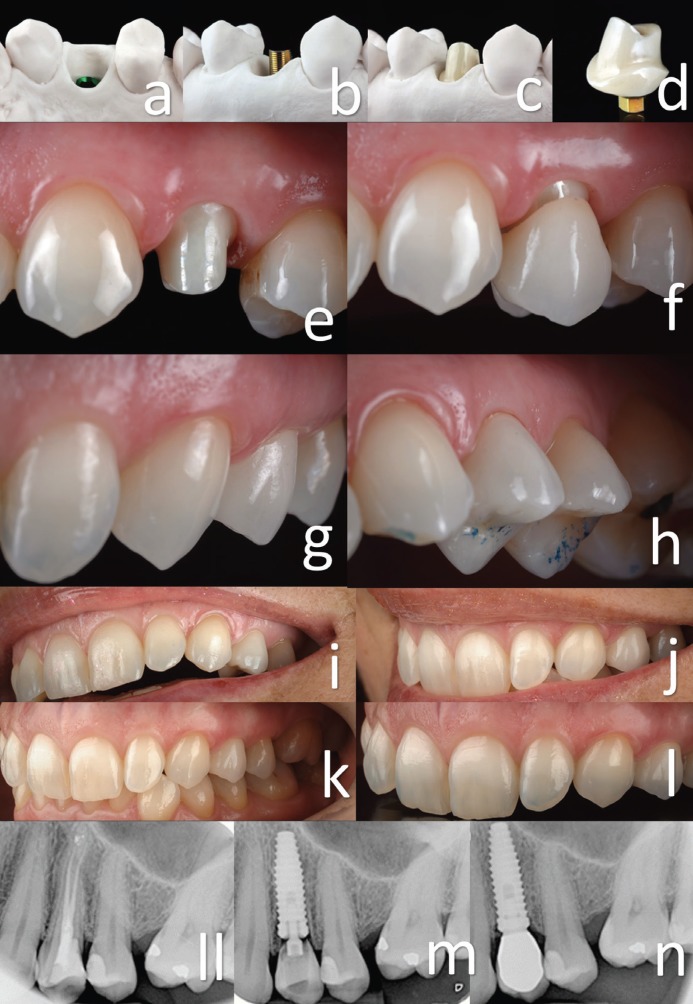
a) Cast with information transferred that faithfully reproduces the emergence profile. b) Testing the metallic interface on the implant analogue. c) Testing the zirconia abutment on the metallic interface. d) Zirconia abutment cemented on the metallic interface. e) Zirconia abutment test for the first time. f) Zirconia crown test for the first time. g) Harmonic results between the pink and white portions of the smile were achieved. h) Checking the occlusion. i, j, k, l) Final result achieved with only three appointments. ll, m, n)Radiographic sequence: initial situation, with the provisional, and with the final restoration.

Six weeks after implant placement, the prosthodontics phase began (Fig. 2e). Using the impression taken in the surgery area, a custom transfer was made with low contraction lab pattern resin (Duralay II, Dental MFG, USA) to verify that the soft tissue’s three-dimensional shape had not changed (Fig. 2f) (12). Reproducing the emergency profile at the same time the temporary restoration is placed guarantees maintenance of the emergency profile and contours gingiva preservation (Fig. 2f) (12,13). Normally, is not necessary to reline the temporary restoration because of receptacle preservation by connective tissue grafting (12). An open-tray impression was taken with polyvinyl siloxane (Variotime, Heraeus Kulzer, USA) to transfer the implant position (9,12). The cast around the implant analog was trimmed to form an emergence profile for future restoration (Fig. 3a). The zirconia framework was waxed on a 4.5-mm titanium base (Laser-Lok Titanium Base Abutment, Bio-Horizons, USA). The shape was scanned, and zirconia abutment was obtained (6,12). After glazing, it was luted onto the titanium base with resin cement (Multilink Hybrid Abutment, Ivoclar Vivadent, ZURICH), and the cement remnants were removed with a sharp scalpel (Fig. 3b,c,d). After luting, the zirconia abutment was scanned, and the zirconia crown structure was obtained. The crown was finished with feldspathic porcelain (VITA VM9, Vita Zahnfabrik, GERMANY) on a zirconia structure (6,10,12).

Third step: Crown placement on the implant and follow-up

After disinfection, the zirconia abutment was attached to the implant (Fig. 3e), causing slight compression and blanching of the tissues, which disappeared after approximately 15 minutes. Immediately, the zirconia ceramic was cemented (Fig. 3f) with resin-modified glass ionomer cement (RelyX Luting; 3M ESPE, St Paul, Minn, U.S.A.). The occlusion was adjusted (Fig. 3g, h).

Due to the excellent reproduction of the area in the impression with an individualized impression coping after the completion of the laboratory procedure, a zirconium abutment and crown finished in the third appointment can be placed (Fig. 3i, j, k, l).

In the follow-up, the next clinical parameters were checked: pain, occlusion, prosthesis mobility and fulfilment of the success criteria. Follow-up examinations were performed at baseline and at 12, 24, and 36 months (Fig. 3ll, m, n). The probing depth, modified plaque index, and modified bleeding index were measured on the mesial, distal, buccal, and palatal surfaces of the implants using a periodontal probe (Hu-Friedy PGF-GFS, Hu-Friedy, Chicago, IL, USA) (5). Intraoral digital radiographs (Schick CDR, Schick Technologies, Long Island City, NY, USA) were also obtained at baseline and 12, 24, and 36 months after implant placement (5). Periapical radiographs were taken perpendicularly to the long axis of the implants following a long-cone parallel technique with an occlusal template to measure the marginal bone level and to calibrate the changes in marginal bone height over time (Fig. 3ll, m, n) (5).

## Discussion

One of the major reasons that patients and clinicians choose postextraction implant as treatment to replace a fractured tooth is because it is faster and predictable (2). It also minimizes the risk of losing the implant using the new implant design (Bihorizons Tapered Implant Plus, USA) and under-drilling the implant site. Postextraction implant and immediately fixing a temporary crown is especially helpful for final aesthetic rehabilitation, as it is possible to perform with an anatomic shape that allows an optimal healing process of the tissues (7). An immediate temporary crown also has the advantage of preserving the shape of the soft tissues as well as patients’ well-being and self-esteem (7). Although CAD/CAM technology is helpful, a major benefit of using the same crown tooth is that it can naturally maintain the natural shape, anatomy, and aesthetics in the area to obtain an ideal emergence profile (2-6).

This case report is in agreement with other studies findings that soft-tissue replacement grafts have become a substantial element to increase tissue volume in plastic periodontal and implant surgery (15). Autogenous subepithelial connective tissue grafts are increasingly applied in aesthetic indications like soft tissue thickening, recession treatment, ridge preservation, soft-tissue ridge augmentation, and papilla re-construction (12). For the clinical performance of connective tissue graft harvesting and transplantation, a fundamental understanding of the anatomy at the donor sites and a sound knowledge of tissue integration and revascularization processes are required (13,15). The selective clinical application of different grafts depends on the amount of required tissue, indication, and personal preference of the treating surgeon. One of the main challenges in the future is to volumetrically evaluate and compare the efficacy and long-term stability of soft-tissue autografts and their prospective substitutes (13,15). This case report has shown that even immediate implant placement using same crown extracted teeth is possible to

maintain soft tissue without forgetting that biological limits to papilla tissue height are dictated by the level of periodontal attachment and bone support. Studies have shown that predictable papilla filling adjacent to a natural tooth is approximately 5 mm from the interdental bone height to the contact point (14).

This case report has shown that the benefits of biocompatibility can be obtained only if the soft tissues have direct contact with the zirconia. Therefore, it can be suggested that the biological advantage of the traditional design for zirconia screw-retained restorations is limited (6). Also, connection type influences the clinical longevity of restorations; in particular, internal connections used in this case with secondary metallic components reduces the incidence of complications (8,9).

Case reports in general do not provide strong clinical evidence. Clinical reports may serve as pilot observations, which could lead to well-designed controlled clinical trials.6 Future clinical studies can show the reaction and long-term follow-up of peri-implant soft tissues and bone levels.

## Conclusions

Within the inherent limitations of this case report, it could be suggested that this three-step technique could produce predictable results: a) first step: implant placement and maintain gingiva emergence profile contours and connective tissue grafting, b) second step: reproduce emergence profile, and c) third step: crown placement on the implant and follow-up.

In most cases, the treatment can be finished in just three appointments. In addition, the patient never loses masticatory and aesthetic function. In other words, complete satisfaction is achieved with a short treatment and the final results of the technique. The case presented demonstrates the 3-year follow-up of successful maintenance of periodontal/peri-implant tissue contours, including interproximal bone and papilla using a soft-tissue surgical approach.
